# Identifying the dominant mode of moisture transport during drying of unsaturated soils

**DOI:** 10.1038/s41598-020-61302-w

**Published:** 2020-03-09

**Authors:** Sudhakar M. Rao, Monica Rekapalli

**Affiliations:** 1 Professor, Department of Civil Engineering, Indian Institute of Science, Bengaluru, 560012 India; 2 Research Associate, Department of Civil Engineering, Indian Institute of Science, Bengaluru, 560012 India

**Keywords:** Hydrogeology, Hydrogeology, Civil engineering, Civil engineering

## Abstract

Diffusion of capillary water and water vapor during moisture loss in an unsaturated soil is impeded by the chemical and geometrical interactions between water molecules/vapor and the soil structure. A reduction in moisture content contracts the diffuse and adsorbed water layers in the partly saturated soil and disturbs the connected capillary network for flow of liquid water. With further drying, the dry soil layer expands and moisture is predominantly lost as vapor through continuous air-flow channels. The water-filled capillary network and air-filled channels are moisture conduits during different stages of soil drying. It is important to identify zones of dominant moisture transport and to select appropriate tortuosity equations for correct prediction of moisture flux. Laboratory experiments were performed to determine moisture flux from compacted soil specimens at environmental relative humidity of 33, 76 and 97% respectively. Analysis of the resultant τ - θ (tortuosity - volumetric water content) relations, illustrated the existence of a critical water content (θ_cr_), that delineates the dominant zones of capillary liquid flow and vapor diffusion. At critical water content, the pore-size occupied by the capillary water is governed by the generated soil suction. Generalized equations are proposed to predict tortuosity factor in zones of dominant capillary liquid flow and vapor transport over a wide range of relative humidity (33 to 97%).

## Introduction

Capillary water flow and vapor transport occur during moisture loss from unsaturated soils. Capillary flow transfers water molecules from the soil interiors to the vaporization plane. The vaporization plane defines the boundary between the partly saturated soil and the dry soil layer. Water molecules vaporize at this boundary and escape to the atmosphere through the connected air-voids of the dry soil layer^1,2^. Increase in moisture loss facilitates the growth of dry soil layer leading to dominance of vapor flow. Impediments to moisture movement in a soil arise from chemical and geometrical interactions between water molecules/vapor and soil structure that is captured by the tortuosity (τ) factor^3,4^. Moldrup *et al*.^5^ observed that the tortuosity to liquid flow is strongly related to the surface area of the soils, while tortuosity to gas transport depends on the connectivity of the air-filled pores. Existence of internal and external surface areas in clay soils would cause considerable fraction of the soil water to not participate in capillary flow and lead to differences in critical volume fractions for percolation of air and water^6^. Ghanbarian *et al*.^7^ expressed tortuosity as a power law function of water content, the critical water content and system size. The results of the authors also indicated that pore connectivity and tortuosity must be treated as two separate properties.

An increase in soil surface area leads to retention of a larger fraction of soil-water in the diffuse ion layer. In a saturated soil, the growth of diffuse ion layer constricts the pore voids and makes the flow-path tortuous^3,8^. Besides, diffuse ion layer formation, water molecules are adsorbed on the soil particles by hydrogen bonds and van der Waals attraction^3,8,9^. In an unsaturated soil, reduction in moisture content during drying contracts the thickness of diffuse and adsorbed water layers that disturbs the network of connected water-filled capillaries^3,10,11^. At very low degree of saturation, moisture movement occurs in the vapor phase; the permeability to vapor transport is high, when the air-phase is continuous^2,12^. The connected capillary water network and air voids network are channels of moisture transport during different stages of soil drying. It is hence important to identify the dominant mode of moisture loss and to select appropriate tortuosity equations for correct prediction of Fickian moisture flux (D_v_)^10^ in a drying soil.

The experimental moisture flux is used to delineate the dominant regions of capillary water flow and vapor transport in an unsaturated soil during moisture loss. Generalized equations are proposed to predict tortuosity factor in regions of dominant capillary liquid flow and vapor transport over a wide range of relative humidity (33 to 97%). Comparisons are made between the experimental and predicted moisture flux by considering appropriate tortuosity factor in Fick’s equation.

## Materials and Methods

### Soil description

Representative soil sample was obtained from 1-m deep pit at the Indian Institute of Science Campus. The specific gravity, liquid limit and plasticity index of the representative soil are 2.66, 34% and 16% respectively^13,14^. Grain size distribution of the representative soil^15^ comprised of 52, 25 and 23% of sand, silt and clay sized fractions respectively. The clay fraction of the soil is composed of non-swelling kaolinite. The maximum dry density and optimum moisture content of the representative soil^16^ corresponds to 1.78 Mg/m^3^ and 16% respectively.

### Experimental moisture flux

Saturated K_2_SO_4_, NaCl and MgCl_2_.6H_2_O solutions maintained constant relative humidity (RH) of 97%, 76% and 33% in desiccators. The environmental humidity imposed by the salt solutions induced moisture loss from the compacted soil specimens (cylindrical specimens of 38 mm diameter and 76 mm length). The desiccators containing the saturated salt solution and compacted soil specimens (placed in the upper half) were maintained at constant temperature of 25 °C (±1 °C) in a temperature controller chamber. Moisture flux under known RH gradient were traced for soil dry density - compaction water content combinations of 1.69 Mg/m^3^ - 11% (series A); 1.78 Mg/m^3^ - 11% (series B); and 1.78 Mg/m^3^ - 16% (series C) respectively.

Triplicate specimens of given series (A/B/C) were horizontally placed on porous polypropylene plate in the upper half of a desiccator (Supplementary information section, Fig. A1) containing the desired saturated salt solution (K_2_SO_4_, NaCl or MgCl_2._6H_2_O). Nine specimens (Triplicates × 3 humidity) of a series were tested at the three humidities.

During drying, the bulk (soil solids + water) weight (W_t_) of the soil specimens were periodically monitored up to 56 days as they negligibly changed thereafter. The gravimetric and volumetric water contents of specimens subjected to 56 days of drying are designated as w_f_ and θ_f_ respectively (f-final). Gravimetric moisture loss of 3–6%, 8–13% and 10–14% were observed on drying the soil specimens at RH of 97, 76 and 33% for 56 days. Slight reductions in porosity (3–6%) and void ratio by (4–9%) occurred after 56 days. The compacted soil specimens subjected to 56 days of drying are termed as desiccated specimens.

The experimental moisture flux (q_vexpt_, g/m^2^/day) at t days of evaporation is calculated as:

1 $${{\rm{q}}}_{{\rm{v}}({\rm{expt}})}=\frac{{{\rm{W}}}_{{\rm{initial}}}-{{\rm{W}}}_{{\rm{t}}}}{{{\rm{A}}}_{{\rm{SA}}}{\rm{t}}}$$

where, W_initial_ is initial mass of soil specimen (t = 0), W_t_ represents the mass of soil specimen after t days of evaporation and A_SA_ is surface area of cylindrical soil specimen (m^2^); The (W_initial_ − W_t_) term represents the loss in gravimetric water content of a compacted specimen on drying at known relative humidity for t days.

The percent variation in W_t_ value of a specimen from the average (three measurements) at any t, ranged between 0.02 to 0.2%. The average W_t_ value of the specimen (range: 148–169 g), at each t, on drying at the desired humidity is utilized in Eq. 1.

Separate batch of A/B/C specimens were tested to obtain SWCC (soil water characteristic curve) plots. The compacted specimens were equilibrated with saturated K_2_SO_4_ (RH = 97%), NaCl (RH = 76%), NaNO_2_ (RH = 64%), MgCl_2_.6H_2_O (RH = 33%) and NaOH (RH = 7%) solutions in desiccators; the bulk weights of the compacted specimens were periodically measured till the specimens experienced negligible weight loss. SWCC plots are obtained for each compaction series by plotting total suction (ψ) as function of final degree of saturation (S_rfinal_). The total suction (ψ) of the specimen was obtained from Kelvin’s equation. The experimental SWCC data is fitted using Fredlund-Xing (FX) equation^17^. The residual water contents (θ_r_) were obtained from the FX curves by using the procedure of Fredlund *et al*.^18^ and correspond to 0.0217, 0.0197 and 0.0287 for Series A, B and C respectively.

### Mercury intrusion porosimetry (MIP) experiments

Pore size distribution of series A/B/C specimens that were dried for fifty-six days at RH of 97%, 76% and 33% were determined using Quanta chrome (USA) Poremaster - 60 over the pressure range of 0.2 to 60000 psi (pounds per square inch). Sample cells having volumes of 0.5 cm^3^ and 2.0 cm^3^ were used in the tests. Prior to performing the MIP test, the specimens were freeze-dried using a lyophilizer. The MIP test was performed in two steps: a low-pressure step from 0.2 psi (1.38 kPa) to 30 psi (206.8 kPa) and a high-pressure step from 20 psi (138 kPa) to 60000 psi (413685 kPa).

## Results

### *τ*_*calc*_*- θ* relation

Utilizing the experimental moisture flux [q_v_(t), Eq. 1], the moisture diffusion coefficient [D_v_ (m^2^/day)] is obtained as:

2 $${{\rm{q}}}_{{\rm{v}}({\rm{t}})}=-\,{{\rm{D}}}_{{\rm{v}}}{{\rm{\rho }}}_{{\rm{vsat}}}\frac{\nabla {\rm{RH}}}{{\rm{R}}{\rm{H}}{\prime} }$$

In Eq. 2, ∇RH is the relative humidity difference between environment (RH_env_ is the RH of the saturated salt solution in the desiccator) and soil pores (RH_soil_), RH′ refers to the average relative humidity of environment and soil pores and ρ_vsat_ is the saturated vapor density (22.99 g/m^3^ at 298 K).

From a knowledge of D_v_ , the tortuosity factor (τ_calc_) at evaporation time t is calculated as:

3 $${{\rm{D}}}_{{\rm{v}}}={{\rm{n}}}_{{\rm{a}}}{{\rm{D}}}_{{\rm{o}}}/{\tau }_{{\rm{calc}}}$$

where, D_o_ (m^2^/s) is the diffusion coefficient in the absence of soil matrix and n_a_ is the air-filled porosity at evaporation period, t.

**Figure 1:**
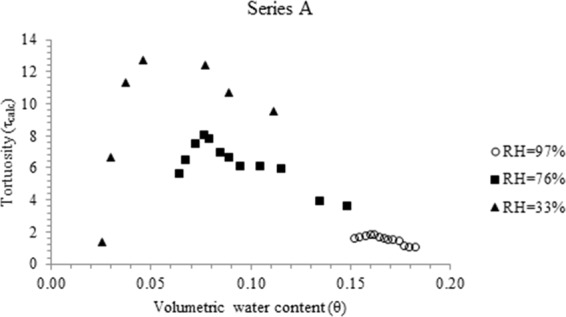
τ_calc_ versus θ plots for series (**A**) specimens exposed to environmental RH of 97%, 76% and 33%.

Figure 1 plots τ_calc_ - θ relations of series A specimens that were exposed to environmental RH of 33, 76 and 97% for various evaporation periods (5–56 days). The τ_calc_ - θ relations of series B and C specimens are provided in the Supplementary information section (Figs. A2 and A3). Up to a critical water content (θ_cr_), a reduction in volumetric water content increases tortuosity (τ_calc_); thereafter, a decrease in volumetric water content (θ) or an increase in volumetric air content (θ_a_) reduces tortuosity.

**Figure 2:**
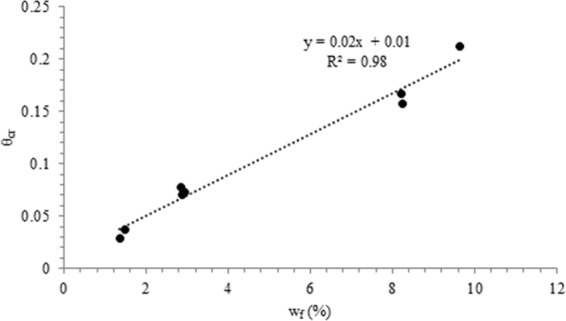
Variation of θ_cr_ with w_f_ for series (**A**–**C**) specimens.

A relation is developed between θ_cr_ and w_f_ (Fig. 2):

4 $${{\rm{\theta }}}_{{\rm{cr}}}=0.02{{\rm{w}}}_{{\rm{f}}}+0.01({{\rm{R}}}^{2}=0.98)$$

Knowing w_f_, the θ_cr_ of a soil experiencing moisture loss is obtained from Eq. 4.

**Figure 3:**
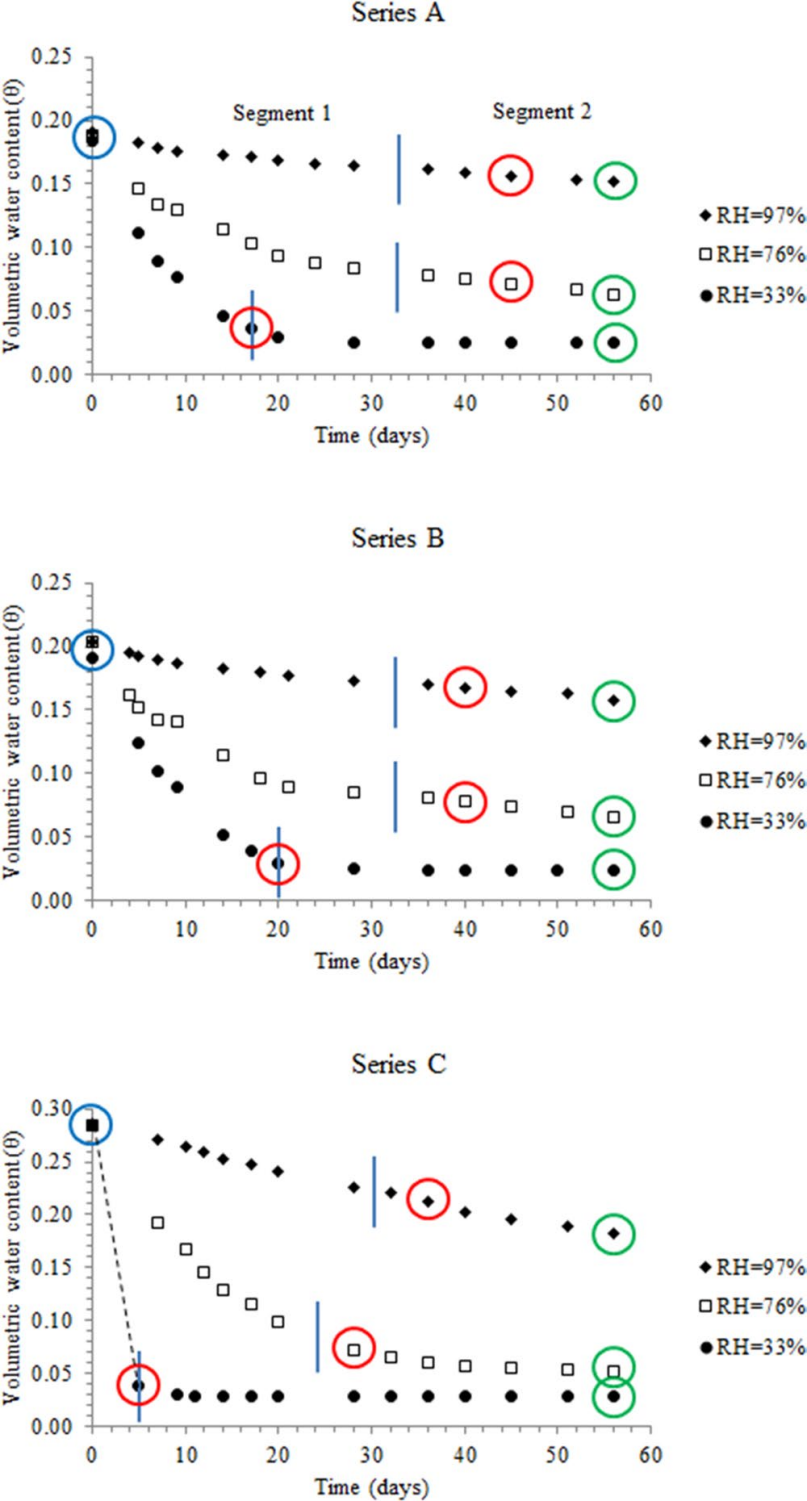
(**A–C**) Variation of volumetric water content with time: Blue, red and green circles refer to initial, critical and final volumetric water contents.

The final water contents are attained by the compacted specimens after long periods of drying (10 to 50 days, Fig. 3). In comparison, moist powder soils dry quickly and attain final water contents in 1 to 5 days^19^. The final water contents of the powder and compacted specimens exposed to given RH (33 to 97%) are near similar (Fig. A4) and follow the equation:

5 $${{\rm{w}}}_{{\rm{f}}({\rm{compacted}})}=1.02{{\rm{w}}}_{{\rm{f}}({\rm{powder}})}-0.024({{\rm{R}}}^{2}=0.98)$$

The w_f_ values of compacted specimens can be quickly determined by testing moist powder specimens and employing it in Eq. 4 to obtain θ_cr_

## Discussion

### Critical volumetric water content *(θ*_*cr*_)

Re-engaging with Fig. 1, it is probable that in the θ > θ_cr_ region, liquid water molecules escape through the connected capillaries of the partly saturated soil. The critical water content signifies the minimum water content for the existence of inter-connected water filled pores and agrees with continuum percolation model^7,20,21^ and the soil-physics concept of a residual water content^22,23^. In θ > θ_cr_ region, the progressive contraction of the diffuse ion layer and adsorbed water layer thickness in soil capillaries contribute to the loss of water-filled pore connectivity. In θ < θ_cr_ region, moisture loss predominantly occurs as vapor diffusion, causing dependence of τ on air-filled porosity.

The θ versus evaporation period (t) plots (Fig. 3) illustrate that the rate of moisture loss is characterized by falling rate segment (segment 1), which results, as the evaporation demand is higher than flow capacity through the connected liquid network^1,2^. The falling rate segment is tailed by near stationary segment (segment 2), wherein, the rate of moisture loss (slope) is very small. The θ_cr_ values are located in segment 2, implying that vapor transport predominates in this segment. The θ_cr_ values have near similar magnitudes as the θ_f_ values (volumetric water content after 56 days of evaporation), which support the development of the relation between θ_cr_ and w_f_ (Fig. 2).

The θ_th_ - soil surface area relation of Moldrup *et al*.^5^ gave a θ_th_ of 0.13 (surface area of soil in this study is 9.78 m^2^/cm^3^), which is close to the lower range of θ_cr_ values at RH = 97% (0.16 to 0.22). The relation of Moldrup *et al*.^5^, however, does not consider the influence of initial soil properties (porosity, water content) or environmental humidity in the determination of θ_th_.

Moldrup *et al*.^5^ and Ghanbarian *et al*.^7^ have recognized that tortuosity increases with reduction in soil water content as the water films surrounding the soil particles become increasingly discontinuous and viscous. At certain threshold soil water content (θ_th_), complete breakage in the continuity of water films causes the liquid phase impedance factor (f) to become zero or the tortuosity factor (τ = 1/√f) to become very large^5^. At θ < θ_th_ values the results of Moldrup *et al*.^5^ show that the tortuosity becomes excessively large and tend towards infinity. This would in principle be true, if capillary flow was the only mode of transport in a drying soil, because then, the actual flow path would become infinitely long at critical water content and beyond^24^. However, in the θ < θ_th_ region where vapor transport dominates, the effective path length should reduce from the participation of connected air-voids in vapor transport. Ghanbarian *et al*.^7^ observed that the sample size is not infinitely large in many cases and the finite size effects dominate the simulated results. Further, when the tortuous path length exceeds the sample length, the finite size scaling is an appropriate approach to generate tortuosity predictions.

The θ_cr_ values ranged between 0.16 to 0.22 on exposure of series A/B/C specimens to environmental RH of 97%. It ranged between 0.077 to 0.1 when the specimens were exposed to environmental RH of 76% and between 0.04 to 0.05 on exposure to environmental RH of 33%. Ghanbarian *et al*.^7^ have observed that critical water contents are not universal but are dependent on the pore structure of the soil.

**Figure 4:**
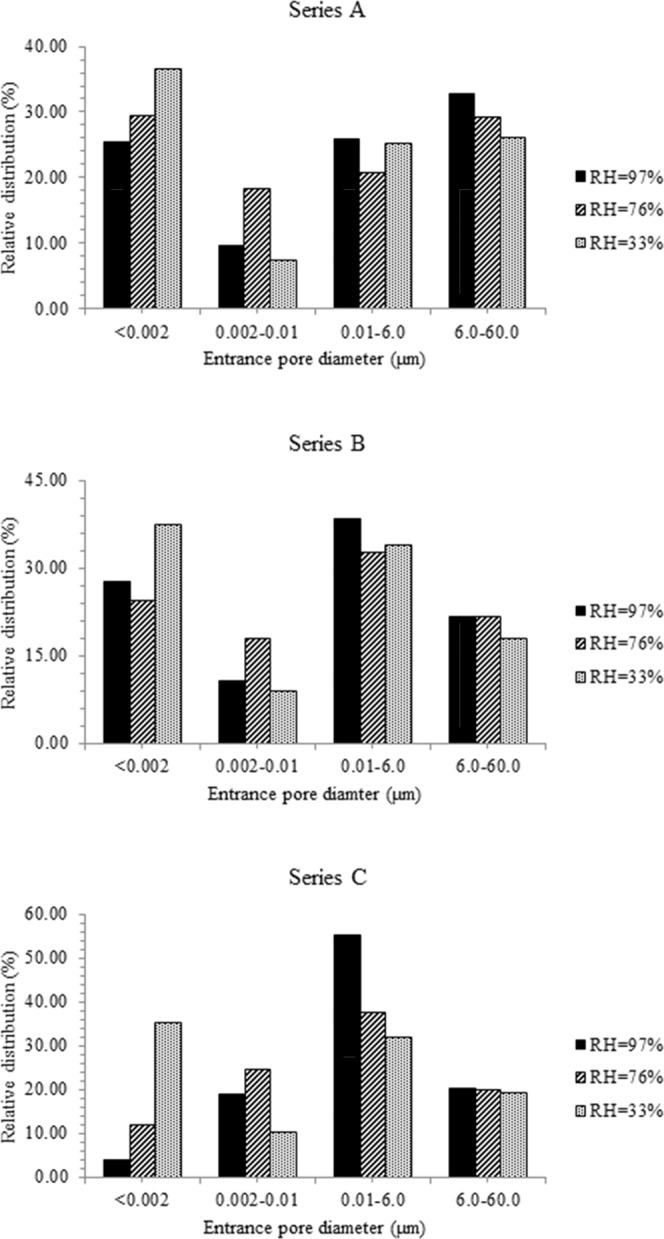
(**A–C**) Frequency distribution plots of desiccated specimens.

The coarse (60 to 6 μm), medium (6 to 0.01 μm), fine (0.01 to 002 μm) and very fine pores (<002 μm) contents^19,25^ contribute to the porosity of compacted specimens after 56 days of drying (desiccated specimens) at the three relative humidities (Fig. 4). The unit volume of voids in the desiccated, A, B and C specimens correspond to 0.213, 0.185 and 0.185 cm^3^/g respectively. Calculations show that for specimens exposed to 97% RH, 60% of the medium pores and all coarse pores are occupied by capillary water at θ_cr_ (0.16–0.21). With soil specimens exposed to RH of 76%, 80% of medium pores are occupied by capillary water at the critical volumetric water content (0.071–0.078). Similarly, for specimens exposed to RH of 33%, all fine pores are occupied by capillary water at θ_cr_ (0.029–0.039). At the critical water content, occupancy of narrower pores by capillary water at the lower RH is commensurate with the larger suction developed by these specimens after 56 days of drying^19^. Hunt^6^ had observed the minimum water content for continuous network of capillary flow in clay soils can be assumed to be 1/6n (n = porosity), plus the volume of pores that are smaller than 0.3 μm radius.

### *θ* > *θ*_*cr*_ condition

The θ - t relations (Fig. 3) depict the dependence of θ on relative humidity (series A/B/C), initial porosity (Series A and B specimens) and gravimetric water content (Series B and C specimens). A normalized volumetric water content (∅):

6 $$\varnothing =\frac{{\rm{\theta }}-{{\rm{\theta }}}_{{\rm{r}}}}{{{\rm{\theta }}}_{{\rm{s}}}-{{\rm{\theta }}}_{{\rm{r}}}}$$

would compensate for variations in initial porosity and water content of dis-similarly compacted soil specimens^18^. In Eq. 6, θ_s_ is the saturated water content (θ_s_ = n) and θ_r_ is the residual water content (θ_r _< θ_f_); the residual water contents are obtained from the SWCC plots (Section 2.2). At θ_r_, the wate_r_ phase is discontinuous and exists as thin water films surrounding the soil particles^26^. The τ - ∅ relations (Supplementary information section, Figs. A5–A7) of compacted specimens (series A, B, C) exposed to similar humidity (97/76/33%) follow equations:

7 $${\rm{\tau }}={\rm{a}}{\varnothing }^{-{\rm{b}}}({\rm{General}}\,{\rm{equation}})$$

8 $${\rm{\tau }}=0.15{\varnothing }^{-2.71}({{\rm{R}}}^{2}=0.98)({\rm{RH}}=97 \% )$$

9 $${\rm{\tau }}=0.81{\varnothing }^{-1.22}\,({{\rm{R}}}^{2}=0.75)({\rm{RH}}=76 \% )$$

10 $${\rm{\tau }}=7.11\,{\varnothing }^{-0.24}({{\rm{R}}}^{2}=0.74)({\rm{RH}}=33 \% )$$

where *a* and *b* are empirical constants at given RH. The trend of *a* parameter suggests that it is related to the rate of desaturation of voids in segment 1 as they also exhibit progressively steep slopes with reduction in RH (Fig. 3). The variations of the empirical constants (*a* and *b*) with RH facilitate determination of τ at any RH of the compacted specimens from the equations:

11 $${\rm{a}}=17.96{({\rm{RH}})}^{2}-34.27({\rm{RH}})+16.46[{\rm{RH}}\,{\rm{in}}\,{\rm{decimals}}]$$

12 $${\rm{b}}=-\,7.51{({\rm{RH}})}^{2}+5.90({\rm{RH}})-1.37[{\rm{RH}}\,{\rm{in}}\,{\rm{decimals}}]$$

### *θ* < *θ*_*cr*_ condition

A normalized volumetric air content for θ < θ_cr_ condition is proposed:

13 $${\varnothing }_{{\rm{a}}}=\frac{{{\rm{\theta }}}_{{\rm{a}}}-{{\rm{\theta }}}_{{\rm{r}}}}{{{\rm{\theta }}}_{{\rm{cr}}}-{{\rm{\theta }}}_{{\rm{r}}}}$$

The ∅_a_ term compensates for variability in volumetric air content of compacted specimens in the θ < θ_cr_ region. Variations of τ with ∅_a_ of the compacted specimens (series A, B, C) exposed to similar humidity (97/76/33%), depicted a reduction in τ with increase in ∅_a_; however, the data set of each compaction series (A/B/C) plot separately. At given RH, the inability of specimens to plot uniquely suggests that the ∅_a_ term is insufficient to characterize vapor phase tortuosity for variable initial porosity and water content conditions. Ghanbarian and Hunt^21^ have used relative air-filled porosity as component of universal scaling law for gas diffusion. In the present study, the universal scaling law could explain the variation of τ of compacted specimens belonging to a single series exposed to given RH. However, like ∅_a,_ the data set of each compaction series (A/B/C) plot separately (do not plot uniquely) at a given RH.

Besides the availability of connected air-voids (∅_a_), the spontaneity of the water vapor to partition between the dry soil layer and atmosphere may contribute to the ease of vapor transport. The distribution coefficient term, K_c_, represents the affinity of a chemical compound to partition between two phases^27^. It could account the partitioning tendency of the water vapor between the dry soil layer and atmosphere. In the present context, the distribution coefficient is defined as:

14 $${{\rm{K}}}_{{\rm{c}}}=\frac{{C}_{s}}{{C}_{e}}$$

where, C_s_ represents the mass (g) of moisture lost per 100 g of soil at given relative humidity and temperature, while, C_e_ is the mass of water remaining in 100 g of soil at evaporation time t. At given t, K_c_ is inversely related to RH (Fig. A8, Supplementary information section). The greater spontaniety of the dry soil layer to lose vapor stems from the more negative change in free energy (∆G°) associated with evaporation at lower RH^19^. Hence the ratio of ∅_a_/(K_c_)^1/RH^ is expected to represent the combined influence of normalized air-filled porosity and RH dependent partitioning tendency of water vapor in the dry soil layer.

The τ - ∅_a_/(K_c_)^1/RH^ relations (Supplementary information, Figs. A9–A11) of compacted specimens (series A/B/C) exposed to similar humidity (97/76/33%) follow the general equation:

15 $${\rm{\tau }}={\rm{x}}{(\frac{{\varnothing }_{{\rm{a}}}}{{{\rm{K}}}_{{\rm{c}}}^{1/RH}})}^{{\rm{y}}}$$

where *x* and *y* are empirical constants. Variations of *x* and *y* with RH follow the polynomial equations:

16 $${\rm{x}}=173.75\,{({\rm{RH}})}^{2}+305.58\,({\rm{RH}})+133.14\,[{\rm{RH}}\,{\rm{in}}\,{\rm{decimals}}]$$

17 $${\rm{y}}=-10.26\,{({\rm{RH}})}^{2}+12.56\,({\rm{RH}})-1.43\,[{\rm{RH}}\,{\rm{in}}\,{\rm{decimals}}]$$

## Validation of Concept

The predictive ability of Eqs. 7, 11 and 12 (θ > θ_cr_) and 15–17 (θ < θ_cr_) is verified by comparing the experimental moisture flux of the compacted specimens exposed to RH of 97/76/33% (Eq. 1) with the predicted moisture flux (Eq. 2). The Eqs. (11, 12 or 16, 17) facilitated calculation of τ at given RH (Eqs. 7 and 15). The τ_calc_ value specified D_v_ (Eq. 3) which in turn provided q_v(pred)_ at different t values (Eq. 2). The goodness of fit of q_v(pred)_ with q_v(expt)_ is obtained by calculating mean relative percentage of deviation modulus (P), given as^28^:

18 $${\rm{P}}( \% )=\frac{100}{{\rm{N}}}\mathop{\sum }\limits_{{\rm{i}}=1}^{{\rm{N}}}\frac{|{{\rm{q}}}_{{\rm{v}}({\rm{expt}}){\rm{i}}}-{{\rm{q}}}_{{\rm{v}}({\rm{pred}}){\rm{i}}}|}{{{\rm{q}}}_{{\rm{v}}({\rm{expt}}){\rm{i}}}}$$

where q_v(expt)i_ and q_v(pred)i_ are experimental and predicted moisture vapor flux of the soil at given RH and time t, and N is number of q_v(expt)_ values measured at various t.

For θ > θ_cr_ condition the compacted specimens mostly exhibit P values of <10% over a wide range (97–33%) of relative humidities (Table 1). For θ < θ_cr_ condition and at RH of 97 and 76%, the P values range between 14–31% with majority of the values varying between 14–19%. Much larger P values are observed at 33% RH (34, 34, 52%). The linear forms of τ - ∅_a_/(K_c_)^1/RH^ relations (not presented) of the compacted specimens are characterized with slopes of 0.26 and 26 at RH of 97 and 33% respectively. The 100-fold variation in slopes indicate that τ is sensitive to small variations in ratio at low relative humidity. The K_c_ values of the compacted specimens range from 0.22 to 0.57 at RH = 97% and from 3.83 to 8.6 at RH = 33%. Possibly, the more spontaneous nature of moisture loss at low RH (large K_c_ values), renders the vapor phase tortuosity sensitive to slight variations in air-filled porosity, leading to higher P values. Ghanbarian and Hunt^21^ have observed that the universal scaling exponent is very sensitive to the measured experimental values at low air-filled porosities.

**Table 1:** Goodness of fit for moisture flux prediction based on proposed equations.

	A-97%	A-76%	A-33%	B-97%	B-76%	B-33%	C-97%	C-76%	C-33%
Eqs. 7, 11, 12 (θ > θ_cr_)	9%	19%	6%	6%	13%	6%	10%	20%	No data points in θ > θ_cr_ region
Eqs. 15–17 (θ < θ_cr_)	16%	17%	34%	19%	14%	34%	17%	31%	52%

Figure 3 reveals that at 33 and 76% RH, bulk (91–97%) of the initial moisture evaporates in the θ > θ_cr_ region. Hence, for soils characterized by falling rate and stationary rate segments that are exposed to evaporation at RH ≤ 76%, considering moisture loss in the θ > θ_cr_ region may be sufficient, as contribution from the θ < θ_cr_ region to overall moisture loss is small.

The P values of series A, B and C specimens (Table 2) were calculated using τ based on Penman^29^, Fredlund *et al*.^18^ and Moldrup *et al*.^30^ equations. Besides τ, all other parameters for moisture flux prediction (Eq. 2) remain the same. Use of Fredlund’s, Penman’s and Moldrup’s equations give P values ranging 42 to 328%, 28 to 102% and 97 to 100% respectively (Table 2). The improved P values from equations proposed in this study (Table 1) underlines the importance of identifying the dominant regions of capillary water flow and vapor transport in unsaturated soils experiencing moisture loss for correct moisture flux prediction.

**Table 2:** Goodness of fit for moisture flux prediction using τ based on other equations.

Reference and relation / RH	Series A			Series B			Series C		
	97%	76%	33%	97%	76%	33%	97%	76%	33%
Fredlund *et al*.^18^ τ = (n_a_)^2/3^	51	162	328	77	56	216	95	40	59
Penman^29^ τ = 0.66*(n_a_)	81	28	102	92	40	65	98	76	82
Moldrup *et al*.^30^ τ = H(θ_s_)^2^[(ψ_e_/ψ)^2/b^ − (ψ_e_/ψ)^1/b^)(θ_th_/θ_s_)]	98	98	98	99	99	97	99	98	100

## Conclusions

The critical water content θ_cr_, separates dominant regions of capillary water flow and vapor diffusion during moisture loss in an unsaturated soil. At θ > θ_cr_ condition, the capillary flow of water molecules dominates moisture loss. When θ becomes less than θ_cr_, vapor diffusion through the air-filled pores of the dry soil layer is important. Both, θ_cr_ and w_f_ occur in the stationary rate segment of the drying curve and have near similar magnitudes. These resemblances encouraged estimation of θ_cr_ from w_f_ values. Specimens exposed to RH of 97, 76 and 33% are constrained to occupy progressively narrower pores at the critical water content owing to larger suction developed in the specimens upon drying. The normalized water content (∅) accounts for the influence of variable initial water content and porosity on capillary water flow tortuosity in the θ > θ_cr_ region. Comparatively, the ∅_a_/(K_c_)^1/RH^ ratio represents the influence of variable volumetric air content and vapor partitioning tendency on vapor phase tortuosity in the θ < θ_cr_ region. The more spontaneous nature of moisture loss at low RH, renders the vapor phase tortuosity sensitive to small variations in air-filled porosity, leading to larger deviations between experimental and predicted moisture flux at RH = 33%. For soils characterized by falling rate and stationary rate segments on exposure to RH ≤ 76%, considering moisture loss only in the θ > θ_cr_ region may be sufficient; this is so, as the contribution from vapor transport in the θ < θ_cr_ region to overall moisture loss is small (3–9% of initial volumetric water content).

## Supplementary information

Supplementary Information.
