# Supplementary material for: Identifying the dominant mode of moisture transport during drying of unsaturated soils
Source: Sci Rep. 2020 Mar 9;10:4322. doi: 10.1038/s41598-020-61302-w (PMC7063045; doi:10.1038/s41598-020-61302-w)
Supplement: Supplementary file 1 — Supplementary Information. [file 41598_2020_61302_MOESM1_ESM.docx]

**Supplementary Information**

Identifying the dominant mode of moisture transport during drying of unsaturated soils

Sudhakar M Rao (corresponding author)

Professor, Department of Civil Engineering, Indian Institute of Science, Bengaluru 560012, India

Email: msrao@iisc.ac.in

Ph: +91 80 2293 2812

Monica Rekapalli

Research associate, Department of Civil Engineering, Indian Institute of Science, Bengaluru 560012, India

Email: [rekapalli.monica@gmail.com](mailto:rekapalli.monica@gmail.com)


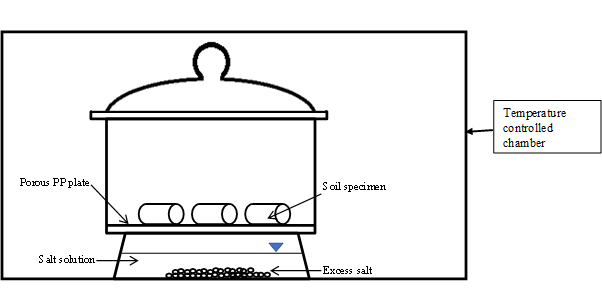


**Figure A1** Schematic of moisture loss experiment (PP: Polypropylene)


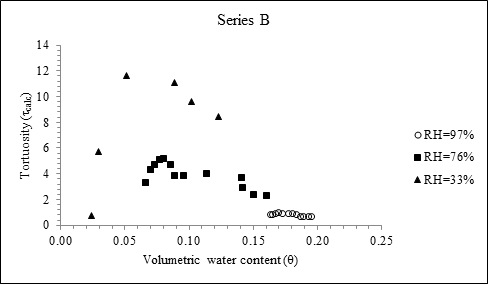


**Figure A2** τ_calc_ versus θ plots for series B specimens exposed to environmental RH of 97%, 76% and 33%


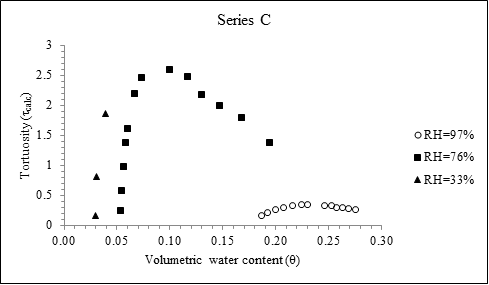


**Figure A3** τ_calc_ versus θ plots for series C specimens exposed to environmental RH of 97%, 76% and 33%


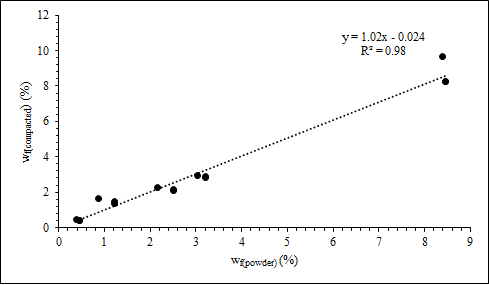


**Figure A4** Variation of w_f(compacted)_ with w_f(powder)_ for series A, B, C specimens


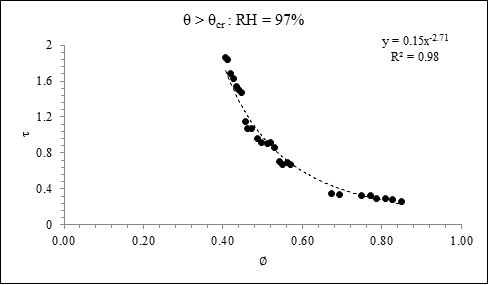


**Figure A5** Variation of τ with ∅ for Series A, B and C specimens at RH = 97%


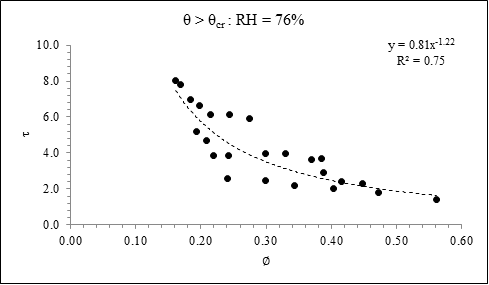


**Figure A6** Variation of τ with ∅ for Series A, B and C specimens at RH = 76%


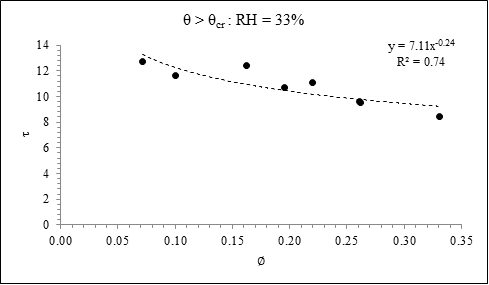


**Figure A7** Variation of τ with ∅ for Series A, B and C specimens at RH = 33%


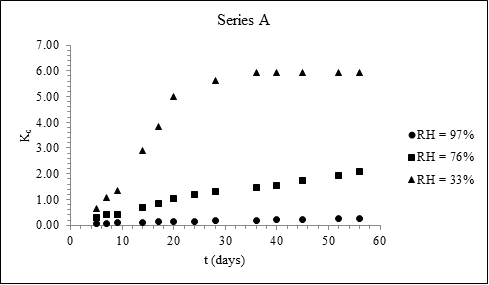


**Figure A8** Variation of K_c_ with time for series A specimens


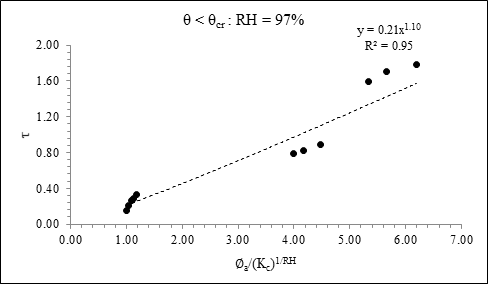


**Figure A9** Variation of τ with ∅_a_/(K_c_)^1/RH^ ratio for series A, B and C specimens at RH = 97%


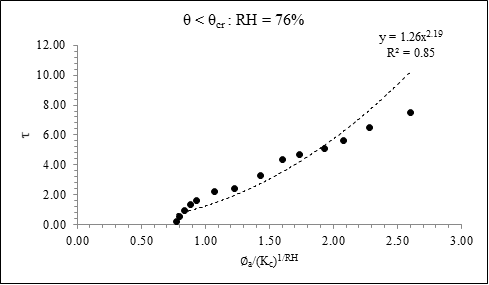


**Figure A10** Variation of τ with ∅_a_/(K_c_)^1/RH^ ratio for series A, B and C specimens at RH = 76%


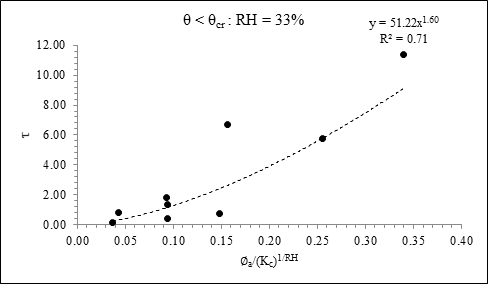


**Figure A11** Variation of τ with ∅_a_/(K_c_)^1/RH^ ratio for series A, B and C specimens at RH = 33%
